# Developmental Reprogramming in Mesenchymal Stromal Cells of Human Subjects with Idiopathic Pulmonary Fibrosis

**DOI:** 10.1038/srep37445

**Published:** 2016-11-21

**Authors:** Diptiman Chanda, Ashish Kurundkar, Sunad Rangarajan, Morgan Locy, Karen Bernard, Nirmal S. Sharma, Naomi J. Logsdon, Hui Liu, David K. Crossman, Jeffrey C. Horowitz, Stijn De Langhe, Victor J. Thannickal

**Affiliations:** 1 Division of Pulmonary, Allergy, and Critical Care Medicine, Department of Medicine, University of Alabama at Birmingham, Birmingham, AL 35294, USA; 2 Heflin Center for Genomic Science, Department of Genetics, University of Alabama at Birmingham, Birmingham, AL 35294, USA; 3 Division of Pulmonary and Critical Care Medicine Department of Internal Medicine, University of Michigan, Ann Arbor, MI 48109, USA; 4 Department of Pediatrics, Division of Cell Biology, National Jewish Health, Denver, CO 80206, USA

## Abstract

Cellular plasticity and de-differentiation are hallmarks of tissue/organ regenerative capacity in diverse species. Despite a more restricted capacity for regeneration, humans with age-related chronic diseases, such as cancer and fibrosis, show evidence of a recapitulation of developmental gene programs. We have previously identified a resident population of mesenchymal stromal cells (MSCs) in the terminal airways-alveoli by bronchoalveolar lavage (BAL) of human adult lungs. In this study, we characterized MSCs from BAL of patients with stable and progressive idiopathic pulmonary fibrosis (IPF), defined as <5% and ≥10% decline, respectively, in forced vital capacity over the preceding 6-month period. Gene expression profiles of MSCs from IPF subjects with progressive disease were enriched for genes regulating lung development. Most notably, genes regulating early tissue patterning and branching morphogenesis were differentially regulated. Network interactive modeling of a set of these genes indicated central roles for TGF-β and SHH signaling. Importantly, fibroblast growth factor-10 (FGF-10) was markedly suppressed in IPF subjects with progressive disease, and both TGF-β1 and SHH signaling were identified as critical mediators of this effect in MSCs. These findings support the concept of developmental gene re-activation in IPF, and FGF-10 deficiency as a potentially critical factor in disease progression.

Idiopathic pulmonary fibrosis (IPF) is a chronic fibrotic lung disease characterized by impaired repair/regenerative responses and aberrant tissue remodeling^1^,^2^. It has been proposed that IPF may represent a re-capitulation of developmental programs based on global genomic studies demonstrating that IPF lungs are enriched with genes associated with lung development, e.g. transcription factors that regulate tissue morphogenesis of embryonic lung^3^,^4^; however, cell-specific expression patterns and the interaction of developmental genes in IPF have not been elucidated. IPF is a heterogeneous disease process with variable clinical courses and some patients are relatively stable for long periods, while others progress more rapidly^5^,^6^,^7^. Factors governing this heterogeneity in disease progression are not well understood.

During early lung development, signals from the mesenchyme are critical to specification of epithelial cell proliferation and differentiation^8^,^9^,^10^. Interactions and signaling between mesenchymal and epithelial cells are critical for later stages of lung development including branching morphogenesis and alveologenesis^11^. Lung branching morphogenesis is regulated by coordinated action of fibroblast growth factor (FGF-10), sonic hedgehog (SHH) and bone morphogenetic protein (BMP-4)^12^,^13^,^14^. Homeobox (Hox) genes are master regulators of tissue patterning and organ development. HoxA1 to A5 and HoxB1 to B6 are expressed in the developing lung^15^. Recently HoxA5 genes have been shown to be key upstream mesenchymal regulators of the Wnt2/2b, one of the main regulators of FGF-10 expression in the lung^16^,^17^. Mesenchyme homeobox-2 (Meox2) regulates TGF-β signaling^18^, nuclear factor-kappa B activity^19^, microRNA-221^20^, and DNA methylation^21^, processes known to be relevant to IPF pathogenesis. Although the precise roles of Hox genes in lung development have not been elucidated, they are known to be expressed at early stages, preceding branching morphogenesis. Roles of these molecules have also been reported in the maintenance of adult lung homeostasis and fibrosis^22^,^23^. FGF-10 is reported to play a major role in alveolar epithelial cell progenitor cell viability^24^,^25^,^26^, and repression of Meox2 is required for TGF-β1 induced myofibroblast differentiation^27^. Thus, dysregulation of these pathways may negatively affect adult lung injury repair.

The participation and contribution of mesenchymal stromal cells (MSCs) to injury repair processes in adult tissues/organs is well recognized^28^. We have previously identified a lung-resident population of MSCs isolated from the lower respiratory tract of human subjects via bronchoscopy and broncho-alveolar lavage (BAL)^29^. BAL-derived MSCs in *ex vivo* culture lack hematopoietic cell markers (CD14, CD34, and CD45), express CD73, CD90, and CD105, and demonstrate the capacity to differentiate into adipocytes, chondrocytes, and osteocytes. These cells were found to be donor-derived up to >11 years (based on sex-mismatch analyses of lung transplant recipients), suggesting that this MSC population is long-lived and reside locally in the terminal airspaces to regulate injury-repair processes^29^. We postulated that these BAL-derived MSCs represent a specific subpopulation of mesenchymal cells that are “embryonic remnants” that lie quiescent within the alveolar interstitium and are mobilized into the alveolar space in the context of lung injury repair. In this study, we hypothesized that gene expression patterns in MSCs from human subjects with varying disease activities/phenotypes may provide clues to aberrant developmental re-programming in IPF. Using differential gene expression and network analyses, we identified central roles for transforming growth factor-β1 (TGF-β1) and sonic hedgehog (SHH) pathways in human subjects with progressive disease; additionally, validation studies indicate a convergence of these pathways on the down-regulation of FGF-10, a critical homeostatic growth factor in alveolar epithelial cell survival and maintenance^24^,^25^,^26^.

## Results

### Gene expression profiling of MSCs in progressive vs. stable IPF

Previous studies from our group demonstrated the presence of tissue-resident MSCs isolated by bronchoscopy and BAL from human subjects^29^. Gene expression profiles in MSCs from IPF have not been previously characterized. To determine the changes in global mRNA expression during IPF progression, MSCs were isolated from patients with stable IPF (s-IPF) and progressive IPF (p-IPF). s-IPF and p-IPF patients were defined by a decline in forced vital capacity (FVC) < 5% and FVC ≥ 10% respectively over the preceding 6 months (n = 4 in each group; protocol for MSC isolation is shown in Supplementary Figure S1). MSCs were characterized, post-isolation and *ex vivo* growth, for their ability to form colonies in tissue culture plates and uniform expression of mesenchymal cell phenotype markers, prolyl-4-hydroxylase and vimentin (Supplementary Figure S2); additionally, we confirmed their ability to differentiate into mesodermal lineages (Supplementary Figure S3). No significant difference was observed in osteogenic, adipogenic and chondrogenic differentiation potential of BAL-derived MSCs between s-IPF and p-IPF MSCs. Early passage MSCs were placed in low serum conditions (0.05%) for 24 hours, then total RNA was isolated and subjected to whole genome transcriptome analysis to determine cellular/molecular mechanisms which separate these distinct clinical subphenotypes. Expression values for each gene using a robust multi-array average were calculated after a number of quality control steps and normalization^30^. As a quality control step, a principal components analysis (PCA) was performed to analyze the segregation of gene expression profiles in each group (Supplementary Figure S4). Based on this PCA, one sample from each group was excluded (sample #3 from s-IPF and sample #8 from p-IPF) resulting in n = 3 in each group for further analysis (GEO repository accession ID # GSE73854).

**Figure 1:**
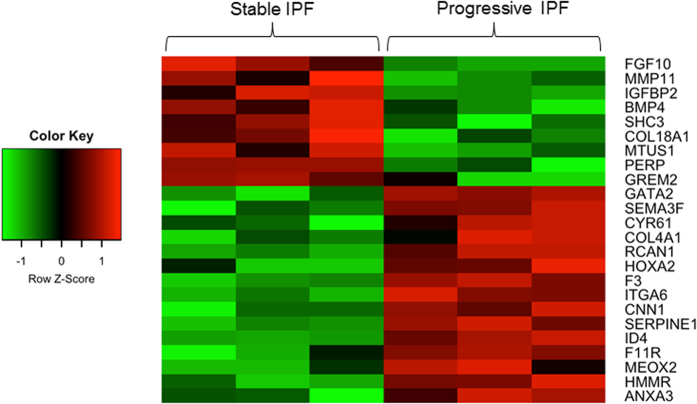
Heat map analysis of lung developmental genes. Heat map representing color coded expression levels of differentially expressed genes in progressive IPF compared to stable IPF (n = 3 in each group; *p* < *0.005*). Up-regulated genes were shown in shades of red whereas down-regulated genes were shown in shades of green.

Statistical analysis was performed fitting a linear model designed for microarray analysis^31^. 428 probesets were selected based on an unadjusted *p*-value less than 0.005. The gene expression data was then analyzed using Ingenuity Pathway Analysis (IPA). The “top functions” predicted by this analysis (score >30; number of molecules ≥20) and associated genes, including those which were up-regulated or down-regulated are shown in Supplementary Table S1. Interestingly, many of the differentially expressed genes are known to play central roles in organismal development (cluster #2 and 5). Among these, the major growth factors that regulate branching morphogenesis during embryonic lung development, FGF-10 and BMP-4 were down-regulated while transcription regulators, Meox2 and HoxA2, were up-regulated in p-IPF when compared to s-IPF. A heat map comprising genes in cluster #2 (with addition of HoxA2 from cluster #5) is shown in Fig. 1, and description of these genes including fold change and p-values are provided in Table 1.

**Figure 2:**
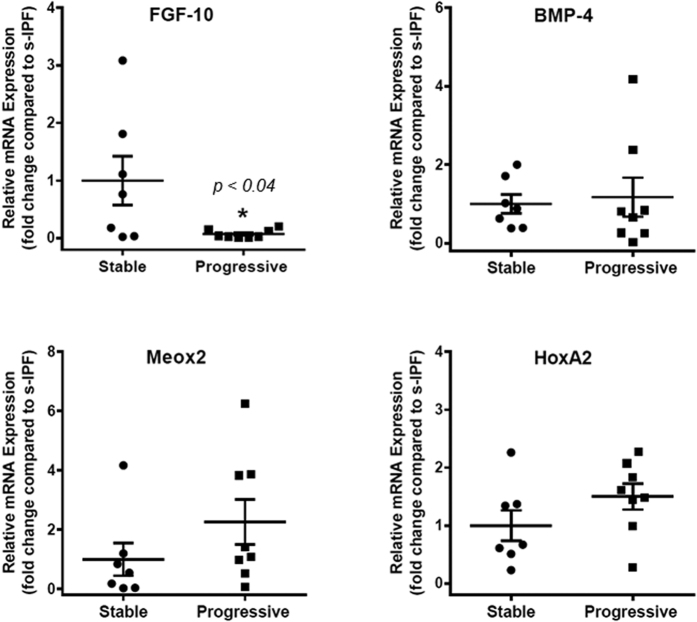
Validation of lung developmental genes of interest. Total RNA was isolated from MSCs from stable IPF (n = 7) and progressive IPF (n = 8), and subjected to real-time PCR analysis for FGF-10, BMP-4, Meox2 and HoxA2. Data were normalized to 18 S rRNA and represented graphically as fold change compared to stable IPF (s-IPF).

To validate the expression of the developmental genes of interest, we analyzed a new cohort of IPF patients (n = 15), including stable-IPF (s-IPF, n = 7) and progressive-IPF (p-IPF, n = 8). Real-time PCR for the genes of interest (FGF-10, BMP-4, Meox2 and HoxA2) was performed on BAL-MSCs on these patients. The primary finding of a down-regulation of FGF-10 in p-IPF was validated with an even greater fold change (−3.77 Log_2_-fold decrease, *p* < *0.04*; t-test) (Fig. 2). To determine if the levels of FGF-10 from IPF subjects is diminished relative to control, non-IPF lungs, we assessed the constitutive expression of FGF-10 mRNA in MSCs obtained from surveillance bronchoscopies and BAL from lung transplant recipients without bronchiolitis obliterans or infection and grown *ex vivo*. We found that steady-state levels of FGF-10 gene expression were >25-fold higher in these non-IPF control subjects (Supplementary Figure S5). BMP-4, Meox2 and HoxA2 showed a trend in the direction of change observed in the original cohort although statistical significance was not achieved (Fig. 2). Together, these data support the concept of the activation of developmental pathways in lung MSCs derived from human subjects with IPF, and a deficiency in MSC expression of FGF-10 in subjects with progressive disease.

### Up-regulation of TGF-β and SHH signaling pathways in progressive IPF

**Figure 3:**
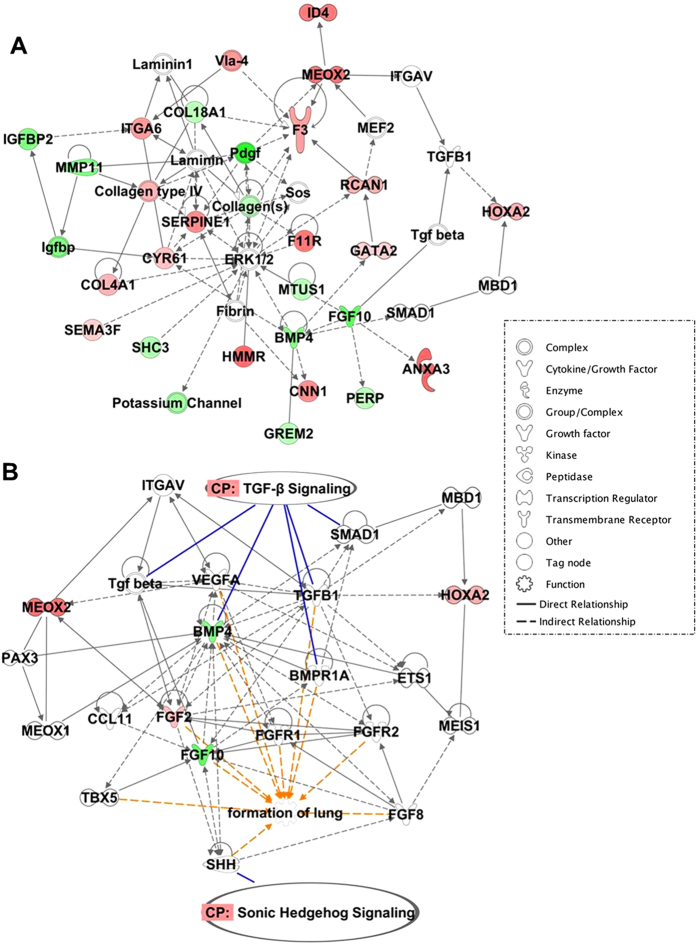
Gene interaction network of organismal development-related genes. (**A**) Ingenuity Pathway Analysis (IPA) was used to generate gene interaction network in progressive IPF. This network contains four developmental genes of interest (FGF-10, BMP-4, Meox2, HOxA2; Table 1). Transcriptional information was projected onto the interaction map such that up-regulated genes are depicted in shades of red and down-regulated genes are in shades of green. (**B**) Shortest path gene interaction network of growth factors (FGF-10 and BMP-4) and transcription regulators (Meox2 and HoxA2). IPA was used to generate this network using their Path Explorer filter which calculates the shortest path between these genes. Blue lines mark the genes found in the canonical transforming growth factor-β (TGF-β) and sonic hedgehog (SHH) signaling; whereas, orange lines indicate genes found to be involved in lung development.

IPA is one approach to identifying the roles of differentially regulated genes in biological pathways/processes^32^. An IPA network analysis of the selected developmental genes, FGF-10, BMP-4, Meox2, and HoxA2, revealed many direct/indirect interactions with other genes within the network that were either up-regulated (red) or down-regulated (green) (Fig. 3A). When “shortest path” analysis between these four genes was performed, two principal biological pathways were uncovered, the canonical TGF-β and SHH signaling pathways (Fig. 3B). These data suggest that both the canonical TGF-β and SHH signaling may participate in developmental programming and contribute to the observed patterns in MSC gene expression.

### TGF-β signaling down-regulates FGF-10, BMP-4, Meox2 and HoxA2 in BAL-MSCs

**Figure 4:**
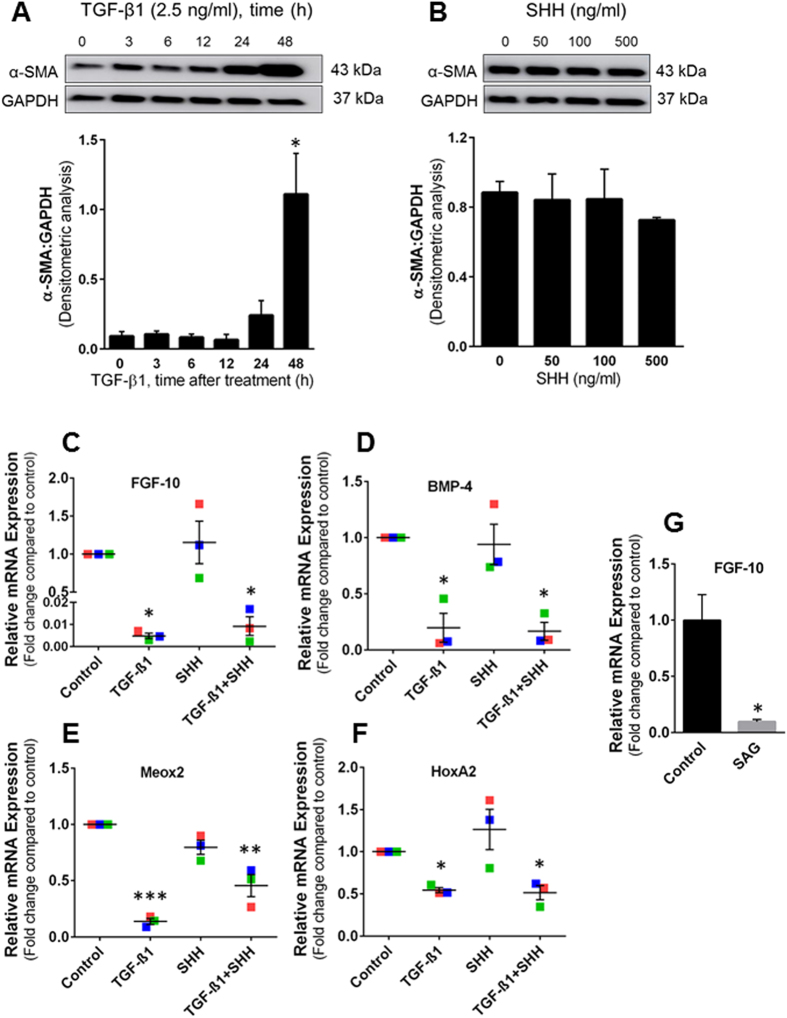
Effects of TGF-β1, SHH and SAG on BAL-derived MSCs. (**A,B**) Myofibroblast differentiation. MSCs were isolated from surveillance bronchoscopies and BAL from lung transplant recipients without bronchiolitis obliterans or infection. MSCs were seeded in 6-well tissue culture plates and serum deprived for 24 h followed by either TGF-β1 treatment (2.5 ng/ml) or SHH (0, 50, 100, 500 ng/ml) for 48 h. Cell lysates were prepared in RIPA buffer and subjected to SDS-PAGE and western blot analysis for α-SMA; GAPDH antibody was used as loading control. Densitometry was performed to quantitate the ratio of α-SMA and GAPDH and plotted graphically; bar graphs represent mean ± SEM, n = 3; *p < 0.05, compared to vehicle treated control. Full-length western blots are presented in Supplementary Figure S6. (**C–F**) RNA expression of developmental genes of interest. Total RNA was isolated from MSCs 48 h post-treatment with either TGF-β1 or SHH or combination and subjected to real-time PCR analysis. Data were normalized to 18 S rRNA and relative mRNA expressions are represented graphically as fold change compared to control. Data represents mean ± SEM; n = 3 (each analyzed in triplicate); *p < 0.01; **p < *0.001;* ***p < 0.0001. Individual colored marker represents average relative mRNA expression of single lung transplant recipient. (**G**) The smoothened agonist, SAG, downregulates FGF-10. MSCs were treated with vehicle and SAG (100 ng/ml) for 48 h; total RNA was extracted and subjected to real-time PCR analysis. Data were normalized to 18 S rRNA and relative mRNA expressions represented graphically as fold change compared to control. Data represents mean ± SEM; n = 3, *p < *0.01*.

Hyperactivation of TGF-β signaling has been implicated in the pathogenesis of IPF including myofibroblast differentiation and survival^33^. Therefore, we first determined whether higher levels of TGF-β are associated with myofibroblast differentiation from BAL-derived MSCs. To test this hypothesis, MSCs were obtained from surveillance bronchoscopies and BAL from lung transplant recipients without bronchiolitis obliterans or infection and grown *ex vivo*. After serum deprived for 24 h, cells were treated with recombinant TGF-β1 (2.5 ng/ml) *in vitro* for 0 to 48 h and expression of α-smooth muscle actin (α-SMA), a marker for the myofibroblast phenotype, was assessed by western blotting. A time-dependent increase in α-SMA was observed in the MSC following TGF-β1 treatment indicating myofibroblast differentiation (Fig. 4A). High levels of SHH have also been reported in IPF lungs^34^. To test if SHH, similar to TGF-β1, induces myofibroblast differentiation of BAL-MSCs, MSCs were serum deprived for 24 h and treated *in vitro* with recombinant SHH (0, 50, 100 and 500 ng/ml) for 48 h and analyzed for α-SMA protein expression. In contrast to TGF-β1, SHH had no effect on α-SMA expression at any of the doses tested (Fig. 4B). These data indicate that TGF-β1, but not the direct actions of SHH, likely contributes to myofibroblast differentiation of MSCs, a key event in fibrogenesis.

Next, we sought to determine whether changes in the pattern of developmental gene expression between s-IPF and p-IPF could be attributed to hyperactive TGF-β or SHH signaling in IPF. MSCs obtained from healthy transplant recipients (n = 3, each analyzed in triplicate) were treated with TGF-β1 (2.5 ng/ml), SHH (500 ng/ml) or in combination for 48 h following 24 h of serum deprivation and assessed gene expression of FGF-10, BMP-4, Meox2 and HoxA2 by real-time PCR. Both TGF-β1 and combination treatment of TGF-β1 with SHH resulted in significant down-regulation of FGF-10, BMP-4, Meox2 and HoxA2 mRNA expression, while SHH by itself did not alter the expression of these genes (Fig. 4C–F). Since exogenous addition of recombinant SHH may fail to bind/activate PTCH1 to de-repress smoothened (SMO; SHH co-receptor), essential for SHH signaling^35^, we tested the ability of a SMO agonist (cell-permeable smoothened agonist, SAG (100 ng/ml; Calbiochem). SAG induced a marked down-regulation in FGF-10 expression in normal human MSCs (Fig. 4G; similar results were obtained in MSCs from a second human subject). SAG had no effect on the expression of BMP-4, Meox2, HoxA2 and α-SMA (data not shown). This data suggests high levels of TGF-β and SHH signaling in IPF may account for the observed deficiency of FGF-10 in human subjects with progressive disease.

### FGF-10 expression is reduced in fibroblastic foci of IPF lungs

**Figure 5:**
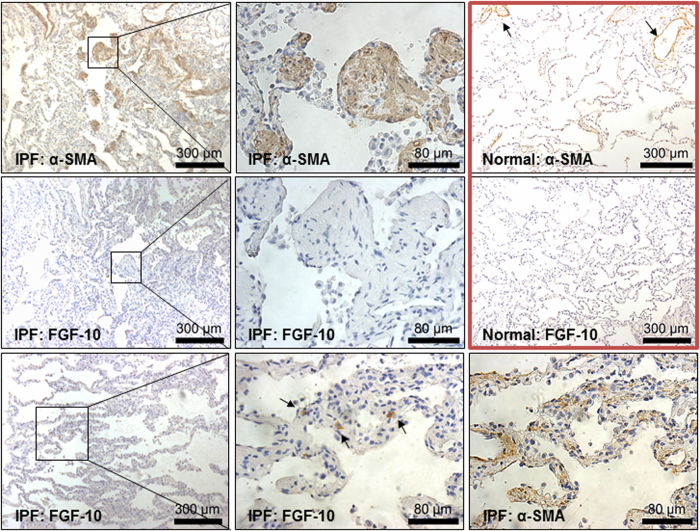
Immunohistochemical localization of α-SMA and FGF-10 in IPF and normal lungs. Six micron (6 μ) sections were obtained from normal (n = 4, failed donor lung) and IPF (n = 4; explant during lung transplantation). Immunohistochemical staining showing expression of the myofibroblast marker, α-SMA, in fibroblastic foci of IPF lung tissues; note that α-SMA staining in normal lung is restricted to large airways and blood vessels (arrows). FGF-10 immunostaining was not detected in the fibroblastic foci although expression of this growth factor was observed in interstitial mesenchymal cells in regions with less fibrotic remodeling (arrows). Higher magnification images of specific regions (boxes) are shown in middle panels.

Based on the finding that FGF-10 expression is decreased in MSCs derived from the BAL of human subjects with progressive IPF, we evaluated the localization of this protein in lungs of control and IPF subjects. Tissue sections obtained from failed donor lungs (controls, n = 4) and IPF subjects (explants from lung transplantation, n = 4) were immunostained for FGF-10 expression. α-SMA immunostaining was also performed to identify areas of active fibrosis. While robust expression of α-SMA was observed within fibroblastic foci in IPF lungs, these foci were largely devoid of FGF-10 expression, despite the expression of this growth factor in interstitial mesenchymal cells in regions with less fibrotic remodeling. We were unable to detect FGF-10 immunostaining in normal lungs and, as expected, only airway/vascular smooth muscle cells stained positive for α-SMA (Fig. 5). IPF lung tissues were also co-immunostained for vimentin, a mesenchymal cell maker, and FGF-10 to determine identity of the FGF-10 producing cells. Immunofluorescence based co-localization demonstrated that almost all FGF-10 positive cells expressed vimentin, while a number of vimentin positive cells failed to express FGF-10 (Supplementary Figure S7). This suggests that FGF-10 expressing cells represent a subset of mesenchymal cells in IPF lung tissues.

## Discussion

Effective lung regeneration following injury requires reactivation of developmental programs which involves the crosstalk between the alveolar epithelium and underlying mesenchymal cells^1^,^36^. In response to lung injury, stromal fibroblasts/myofibroblasts deposit extracellular matrix (ECM) proteins, mainly fibrillar collagens and fibronectin, to form a provisional matrix that allows for alveolar epithelial cell proliferation and differentiation to regenerate damaged epithelium. Chronic injury and aging may exhaust mechanisms by which the mesenchyme and epithelium regenerate functional alveoli and lead, instead, to aberrant mesenchymal activation characterized by myofibroblast accumulation and excessive ECM deposition. The roles of alveolar mesenchymal cells in lung injury repair are not well understood, and likely represent a heterogeneous population^37^. We have previously identified lung-resident MSCs that can be isolated and analyzed by bronchoscopy and BAL^29^.

In the current study, we identified an interesting pattern of gene expression in BAL-derived MSCs from patients with IPF, when differentially analyzed based on a clinical definition of disease progression [forced vital capacity (FVC) decline of ≥10% over a 6-month period] vs. stability (FVC < 5% over a 6-month period). The robustness of this definition of disease progression has been validated by 3 independent research groups that showed that a decline in FVC of ≥10% over a 6–12 month period is predictive of decreased survival in IPF subjects^5^,^6^,^7^. For this study, we relied on early passage (P1–4) MSCs *ex vivo* to maintain relative purity of the mesenchymal cell population (rather than P0 MSCs that are less pure; no specific cell surface marker of MSCs has been reported). Although gene expression patterns are influenced by *ex vivo* cell culture conditions, these cells also maintain a stable and heritable pattern of gene expression, likely via cell autonomous epigenetic mechanisms^38^,^39^. Transcriptomic analyses on MSCs isolated from a discovery cohort of IPF patients with progressive vs. stable disease (n = 4 in each group, and n = 3 per group after PCA) revealed enrichment for genes involved in organismal development. Among these genes, differential expression of genes regulating branching morphogenesis (FGF-10, BMP-4) and the homeobox genes, Meox2 and HoxA2, were identified. Interactome analysis of these four genes by shortest pathway network algorithm indicated their association with canonical TGF-β1 and SHH signaling. We explored whether TGF-β1 and SHH signaling in MSCs were sufficient to explain the observed patterns of developmental gene expression that discriminated progressive from stable IPF. TGF-β1 suppressed FGF-10, BMP4, Meox2 and HoxA2 gene expressions in MSCs. While recombinant SHH had no effect on these genes of interest, treatment of MSCs with an agonist of smoothened (SMO; SHH co-receptor) resulted in inhibition of FGF-10 gene expression without significant effects on BMP4, Meox2 and HoxA2. We re-analyzed these 4 candidate genes in a replication cohort of 15 IPF subjects (8-progressive and 7-stable) and observed an even greater decrease in FGF-10 (−3.77 Log_2_-fold change; *p* < *0.04*) in IPF subjects with progressive disease; the other developmental genes of interest showed similar trends to the discovery cohort, although statistical significance was not achieved. Non-IPF control MSCs obtained from surveillance bronchoscopies and BAL from lung transplant recipients without bronchiolitis obliterans or infection showed significantly higher FGF-10 expression compared to IPF MSCs. This data supports the concept that there may be a loss of a protective effect of FGF-10 in progressive IPF.

Mesenchyme-derived FGF-10 is critical for maintaining lung epithelial progenitor cells during early lung development^40^. FGF-10 knockout mice fail to develop lungs^41^. FGF-10 expression is also required for lung epithelial regeneration following injury in adults, and suppression of FGF-10 has been shown to compromise regenerative capacity after naphthalene injury^42^. FGF-10 acts as a protective and therapeutic agent against bleomycin-induced pulmonary fibrosis^24^ and attenuates H_2_O_2_-induced alveolar epithelial cell DNA damage^26^. FGF-10 overexpression during bleomycin-induced lung injury has been shown to have a protective effect on epithelial progenitor cells by inhibiting TGF-β1^24^. Thus, TGF-β1 and FGF-10 reciprocally and negatively regulate each other to influence the outcome of the repair and regenerative response. Absence of FGF-10 immunostaining in the fibroblastic foci (myofibroblasts) in IPF lungs substantiates TGF-β1-stimulated suppression of FGF-10 gene expression in our *in vitro* studies. A recent report also demonstrated that intra-tracheal delivery of FGF-10 protected from LPS-induced acute lung injury in rats via mobilizing MSCs in the BAL^43^. It remains to be determined whether the mechanism of protection observed in this study is solely related to MSC mobilization or whether this protection may involve mesenchymal-to-epithelial cell signaling. Our study focused on human subjects with progressive (vs. stable) fibrotic lung disease, supporting a role for FGF-10 in disease progression rather than disease initiation or susceptibility.

SHH has been shown to inhibit FGF-10 gene expression in embryonic lung tissue^35^,^44^,^45^. SHH was also reported to stimulate proliferation and myofibroblast differentiation of human lung fibroblasts^34^,^46^. High levels of SHH is also reported in IPF lungs^34^,^47^. SHH expression is also required during embryogenesis and tissue repair^48^,^49^. Recently, SHH was shown to be required for maintenance of a quiescent mesenchyme in the adult lung^50^. During epithelial injury, SHH levels decline leading to a proliferative repair response by mesenchymal cells. SHH levels promptly returns to baseline post injury restoring mesenchymal quiescence^50^. In our study, while exogenous treatment with recombinant SHH failed to suppress FGF-10 expression in MSCs, activation of this pathway by SAG (a smoothened agonist) markedly down-regulated FGF-10 in lung MSCs. The failure of recombinant SHH to mediate signaling may be related to its inability to bind/activate PTCH1 and de-repress SMO, essential for SHH signaling^35^. Together, these data support a role for heightened SHH/smoothened signaling, in concert with TGF-β1, in the downregulation of FGF-10 gene expression observed in MSCs of human IPF subjects with progressive disease.

BMP-4 is principally expressed by the lung epithelial cells and is weakly expressed by the mesenchyme during lung development^51^. Unlike other BMPs, BMP-4 expression remains restricted to the distal epithelial cells and regulates branching morphogenesis along with FGF-10 and SHH^48^,^52^. FGF-10 is known to stimulate BMP-4 expression in the epithelium during branching and in return is inhibited by SHH and BMP-4 at high concentrations, thus controlling branch outgrowth^53^. TGF-β1 has been reported to inhibit BMP-4 signaling in pulmonary artery smooth muscle cells^54^. Similar to this latter report, we observed a significant down-regulation of BMP-4 gene expression by TGF-β1; the precise role for mesenchyme-derived BMP-4 in wound healing and repair requires further investigation.

TGF-β1’s role in fibrotic disorders is well recognized, not only in the lung but in almost all organs studied^55^. Alveolar epithelial cells are thought to be the primary source of TGF-β1 in IPF lungs^56^,^57^. TGF-β1 is known to regulate embryonic development, cellular growth/differentiation, host defense and tissue repair^58^. Our findings showed significant downregulation of FGF-10 gene expression in MSCs in response to TGF-β1 and SHH signaling. The sustained up-regulation of TGF-β1 and SHH, with concomitant deficiency of FGF-10, may prevent restoration of normal epithelial homeostasis.

In summary, this study demonstrates lung-resident MSCs harbor gene expression patterns that recapitulate developmental reprogramming in an adult, age-related fibrotic lung disease, IPF. This is the first study to demonstrate a deficiency in MSC expression of FGF-10 in progressive IPF, a specific clinical endotype. This is consistent with a protective role of FGF-10 in animal models of lung injury and fibrosis^24^,^40^,^43^,^59^. Further studies are required to determine the impact of the FGF-10 deficiency on epithelial cell fate and function. FGF-10^low^ population may be a biomarker of “pathologic” MSCs and that loss of FGF-10 alone is unlikely to explain disease progression. The studies reported here also suggest that gene expression patterns of alveolar MSCs may be useful in prognostication of disease progression in IPF, and potentially other chronic lung diseases; future studies will determine if such approaches can be exploited to develop personalized therapies that target altered developmental pathways.

## Methods

### Isolation of alveolar mesenchymal cells

BAL fluid was collected from patient with stable and progressive IPF following patient informed consent and methods approved by the Institutional Review Board (IRB) of the University of Michigan (UM) and University of Alabama at Birmingham (UAB). All methods involving human participants were performed in accordance with the relevant guidelines and regulations. The cellular fraction of the BAL fluid is pelleted by centrifugation and plated on tissue culture plates at a density of 5 × 10^6^ cells/100 mm dish. The media is changed daily for the first two days and thrice weekly, thereafter. This resulted in the removal of terminally differentiated, apoptotic cells and/or non-adherent cells, mainly inflammatory cell populations (macrophages). By 10–14 days in cell culture, a number of colony forming units of MSCs (CFU-MSCs) are seen on Giemsa staining. Serial dilutions allowed an estimate of the number of clonally expanding cells in the original BAL; for example, in the patient represented, 4 colonies were visualized at 1:1000 dilutions, indicating ~4000 cells in the original 5 × 10^6^ cells (0.08%). MSCs, at passage 1, show uniform staining for prolyl-4-hydroxylase and vimentin, supporting mesenchymal cell phenotype (Supplementary Figure S2). All BAL-MSCs used in this study were between passages 1–4.

### Mesodermal lineage differentiation Assay

For osteogenic differentiation of BAL-derived MSCs, cells were seeded at a concentration of 10^4^ cells/cm^2^ in 24-well culture dishes and allowed to grow for 24 h. The growth medium was replaced with medium containing 10^–8^ M dexamethasone, 0.2 mM ascorbate phosphate, and 10 mM β-glycerophosphate in α-MEM (Invitrogen; Thermo Fisher Scientific, Waltham, MA) with 10% FBS. Medium was changed every 2 days. After 14 days cells were washed, fixed with 10% formalin and stained with Alizarin red stain to visualize calcium deposition. For adipogenic differentiation of MSCs, cells were seeded at a density of 10^4^ cells/cm^2^ in 24-well culture dishes and allowed to grow for 24 h. The MSCs were then exposed to StemPro adipocyte differentiation medium (Thermo Fisher Scientific). Fresh medium was added to the MSCs every 2 days. After 10 days the MSCs were fixed and stained with Oil Red-O stain (SIGMA) to visualize lipid accumulation in the MSCs. Adipocyte nuclei were counterstained with hematoxyllin. For chondrogenesis assay, 3 × 10^5^ MSCs were pelleted in 15 ml conical bottom tubes and cell pellet was exposed to StemPro chondrocyte differentiation medium (Thermo Fisher Scientific) for 14 days. Medium was changed every 2 days. The cell pellets were stained with Safranin-O aqueous staining solution (EMD Millipore, Billerica, MA) for cellular proteoglycans specific to cartilage.

### Affymetrix Gene Chip Analysis

MSCs were grown up to 80–90% confluence; and serum deprived for 24 h at passage 2, which represents the “pooled” colonies. Total RNA was isolated using Qiagen RNeasy Mini Kit (Valencia, CA) and subjected to whole genome transcriptomal analysis. RNA isolates (n = 4 in each group) were hybridized on Affymetrix U133A microarray chips with 22976 probe-pairs and statistical analyses were performed at the University of Michigan Microarray Core Facility.

### Systems Biology analysis

For generating networks, a data set containing gene identifiers and corresponding expression values was uploaded into the Ingenuity Pathway Analysis software (Ingenuity Systems, www.qiagen.com/ingenuity). Each identifier was mapped to its corresponding object in Ingenuity’s Knowledge Base. A fold change cutoff of ±2 and a q-value cutoff of 0.05 were set to identify molecules whose expression was significantly differentially regulated. These molecules, called Network Eligible molecules, were overlaid onto a global molecular network developed from information contained in Ingenuity’s Knowledge Base. Networks of Network Eligible Molecules were then algorithmically generated based on their connectivity. The Functional Analysis identified the biological functions and/or diseases that were most significant to the entire data set. Molecules from the dataset that met the fold change cutoff of ± 2 and a q-value cutoff of 0.05 and were associated with biological functions and/or diseases in Ingenuity’s Knowledge Base were considered for the analysis. Right-tailed Fisher’s exact test was used to calculate a p-value determining the probability that each biological function and/or disease assigned to that data set is due to chance alone.

### *Ex vivo* myofibroblast differentiation

BAL-derived MSCs (P3; 100,000 cells) were plated in each well of 6-well tissue culture dishes in DMEM supplemented with 4.5 g/L glucose, 10% FBS, 1 mM L-glutamine and penicillin-streptomycin. The next day the cells were serum starved for 24 h and subjected to either vehicle or recombinant TGF-β1 (R&D Systems, Minneapolis, MN) or recombinant SHH (R&D Systems) for 48 h to test for myofibroblast differentiation. Proteins were isolated from MSCs post-treatment and subjected to western blotting for α-SMA expression.

### Real-Time PCR

Total RNA was isolated from cells using the RNeasy Mini Kit (Qiagen) and reverse transcribed using iScript Reverse Transcription SuperMix for real-time PCR (Bio-Rad, Hercules, CA). Real-time PCR reactions were performed using SYBR Green PCR Master Mix (Life Technologies, Grand Island, NY) and gene specific primer pairs for FGF-10, BMP-4, Meox2, HoxA2, and 18 S rRNA (Integrated DNA Technologies, Coralville, IA; for primer sequences, see Supplementary Table S2). Reactions were carried out for 40 cycles (95 °C for 15 sec, 60 °C for 1 min for FGF-10, BMP-4 and HoxA2; 95 °C for 15 sec, 66 °C for 1 min for Meox2) in a StepOnePlus Real-Time PCR System (Life Technologies). Real-time PCR data is expressed for each target gene normalized to endogenous 18 S, as 2^−ΔΔCt^ and relative mRNA expression is represented graphically as fold change compared to control.

### Western Blotting

Protein lysates were collected from each time point using RIPA cell lysis buffer supplemented with sodium orthovanadate (SIGMA-Aldrich, St Louis, MO) and protease inhibitor cocktail (EMD Millipore). Protein concentrations were determined using Pierce BCA micro protein assay kit (Thermo Fisher Scientific) and a microplate reader (BioTek Instruments, Winooski, VT). The lysates were then denatured and reduced using 4x Laemmli sample buffer and 10x reducing agent (Life Technologies) at 70 °C for 10 minutes. The proteins were separated on a 4–20% Tris-glycine Bio-Rad Criterion precast gradient gel and subjected to western blotting. Briefly, proteins were transferred to a nitrocellulose membrane using a Bio-Rad transfer apparatus. Membranes were blocked with Superblock T20 (Thermo Fisher Scientific) and probed with antibodies against human α-SMA (American Research Product Inc; Waltham, MA). GAPDH (Cell Signaling, Danvers, MA; dilution 1:1000) was used as a loading control. Signals were detected by probing with anti-mouse secondary antibodies labeled with horse radish peroxidase (Pierce) and immunoblots were developed with Luminata Crescendo Western Blot HRP substrate (EMD Millipore). Blots were imaged in Amersham Imager 600 (GE Health Care, Pittsburg, PA). Density of the protein bands were measured using ImageQuant TL software (GE). Data presented as the mean of three experiments. The error bar represents the standard error of mean.

### Immunostaining of lung tissue sections

Human lung tissues were obtained from patients with confirmed diagnosis of IPF^60^, under protocol approved by the IRB of UM and UAB. Informed consents were obtained from all individuals enrolled through the Airway Tissue Procurement Program at UM and UAB. Normal and IPF lung tissues were fixed in formaldehyde, dehydrated and paraffin embedded using standard protocol. Six micron thick sections were cut and mounted on glass slides and immunostained for human α-SMA (mouse monoclonal; American Research Products; 1:500), FGF-10 (rabbit polyclonal; EMD Millipore; 1:100), and vimentin (mouse monoclonal; SIGMA-Aldrich; 1:100). Briefly, tissue sections were deparaffinized in xylene and hydrated through ethanol series and water. Antigen retrieval was performed using citrate buffer at pH 6.0 in a 95 °C water bath followed by quenching of endogenous peroxidases using 3% hydrogen peroxide. Tissue sections were blocked using 5% normal goat (immunohistochemistry) or donkey serum (immunofluorescence) and were then incubated in primary antibodies overnight at 4 °C. No primary antibody and appropriate IgG isotype controls were utilized to determine specificity of staining. Secondary detection was performed using the Dako Envision Dual Link System (Carpinteria, CA) for α-SMA and FGF-10 (IHC). For immunofluorescence detection of vimentin and FGF-10, anti-mouse/anti-rabbit Alexa Fluor 594/488 (Molecular Probes, Thermo Fisher) secondary antibodies were used. No primary antibody and appropriate IgG isotype controls were utilized to determine specificity of staining. Colorimetric detection was achieved using the DAB/H_2_O_2_ kit from the Vector Laboratories (Burlingame, CA). Nuclei were counterstained with hematoxylin (Vector Labs) for IHC and Hoechst dye (Molecular Probes) for immunofluorescence.

### Statistical Analyses

Statistical analyses and graphical presentations were prepared in GraphPad PRISM version 6.0 software. Western blotting and real-time PCR data obtained in this study were analyzed by unpaired two-tailed Student’s *t*-test for pairwise comparisons. If more than two groups were present, data were analyzed by ANOVA followed by Tukey’s post hoc test for multiple pairwise comparisons. Results were considered significant if *p* < *0.05*.

## Additional Information

**How to cite this article**: Chanda, D. *et al.* Developmental Reprogramming in Mesenchymal Stromal Cells of Human Subjects with Idiopathic Pulmonary Fibrosis. *Sci. Rep.* **6**, 37445; doi: 10.1038/srep37445 (2016).

**Publisher’s note:** Springer Nature remains neutral with regard to jurisdictional claims in published maps and institutional affiliations.

## Supplementary Material

Supplementary Information

## Figures and Tables

**Table 1:** Differentially expressed lung developmental genes in progressive (vs. stable) IPF.

Symbol	Entrez Gene Name	Affymetrix ID	Fold Change (LOG_2_)	*p-value*	Location	Type(s)
Down-regulated genes in progressive IPF						
**BMP4**	Bone morphogenetic protein 4	211518_s_at	−2.58	0.001	Extracellular Space	growth factor
COL18A1	Collagen, type XVIII, alpha 1	209082_s_at	−1.58	0.002	Extracellular Space	other
**FGF10**	Fibroblast growth factor 10	231762_at	−3.23	0.000	Extracellular Space	growth factor
GREM2	Gremlin 2, DAN family BMP antagonist	220794_at	−1.54	0.004	Extracellular Space	other
IGFBP2	Insulin-like growth factor binding protein 2, 36 kDa	202718_at	−2.58	0.000	Extracellular Space	other
MMP11	Matrix metallopeptidase 11	203878_s_at	−2.65	0.001	Extracellular Space	peptidase
MTUS1	Microtubule associated tumor suppressor 1	212095_s_at	−1.56	0.002	Other	other
PERP	PERP, TP53 apoptosis effector	236009_at	−1.55	0.001	Plasma Membrane	other
SHC3	SHC (Src homology 2 domain containing) transforming protein 3	229824_at	−1.75	0.001	Cytoplasm	other
Up-regulated genes in progressive IPF						
ANXA3	Annexin A3	209369_at	3.01	0.000	Cytoplasm	enzyme
CNN1	Calponin 1, basic, smooth muscle	203951_at	2.29	0.000	Cytoplasm	other
COL4A1	Collagen, type IV, alpha 1	211981_at	1.65	0.004	Extracellular Space	other
CYR61	Cysteine-rich, angiogenic inducer, 61	210764_s_at	1.41	0.004	Extracellular space	other
F3	Coagulation factor III (thromboplastin, tissue factor)	204363_at	2	0.000	Plasma Membrane	transmembrane receptor
F11R	F11 receptor	223000_s_at	2.71	0.000	Plasma Membrane	other
GATA2	GATA binding protein 2	209710_at	1.16	0.004	Nucleus	transcription regulator
HMMR	Hyaluronan-mediated motility receptor (RHAMM)	207165_at	2.91	0.000	Plasma Membrane	other
ID4	Inhibitor of DNA binding 4, dominant negative helix-loop-helix protein	209291_at	2.65	0.000	Nucleus	transcription regulator
ITGA6	Integrin, alpha 6	201656_at	2.09	0.000	Plasma Membrane	other
**MEOX2**	Mesenchyme homeobox 2	206201_s_at	2.82	0.001	Nucleus	transcription regulator
RCAN1	Regulator of calcineurin 1	215253_s_at	1.71	0.000	Nucleus	transcription regulator
SEMA3F	Sema domain, immunoglobulin domain (Ig), short basic domain, secreted, (semaphorin) 3 F	206832_s_at	1.2	0.004	Extracellular Space	other
SERPINE1	Serpin peptidase inhibitor, clade E (nexin, plasminogen activator inhibitor type 1), Member 1	1568765_at	2.35	0.000	Extracellular Space	other
**HOXA2**	Homeobox A2	1557051_s_at	1.73	0.002	Nucleus	transcription regulator

## References

[b1] Duffield J. S., Lupher M., Thannickal V. J. & Wynn T. A. Host responses in tissue repair and fibrosis. Annu Rev Pathol. 8, 241–276 (2013). 23092186 10.1146/annurev-pathol-020712-163930 PMC3789589

[b2] Thannickal V. J., Toews G. B., White E. S., Lynch J. P. 3rd & Martinez F. J. Mechanisms of pulmonary fibrosis. Annu Rev Med. 55, 395–417 (2004). 14746528 10.1146/annurev.med.55.091902.103810

[b3] Selman M., Pardo A. & Kaminski N. Idiopathic pulmonary fibrosis: aberrant recapitulation of developmental programs? PLoS Med. 5, e62 (2008). 18318599 10.1371/journal.pmed.0050062 PMC2265304

[b4] Hardie W. D., Glasser S. W. & Hagood J. S. Emerging concepts in the pathogenesis of lung fibrosis. Am J Pathol. 175, 3–16 (2009). 19497999 10.2353/ajpath.2009.081170 PMC2708789

[b5] Collard H. R. *et al.* Changes in clinical and physiologic variables predict survival in idiopathic pulmonary fibrosis. Am J Respir Crit Care Med. 168, 538–542 (2003). 12773325 10.1164/rccm.200211-1311OC

[b6] Latsi P. I. *et al.* Fibrotic idiopathic interstitial pneumonia: the prognostic value of longitudinal functional trends. Am J Respir Crit Care Med. 168, 531–537 (2003). 12791580 10.1164/rccm.200210-1245OC

[b7] Flaherty K. R. *et al.* Prognostic implications of physiologic and radiographic changes in idiopathic interstitial pneumonia. Am J Respir Crit Care Med. 168, 543–548 (2003). 12773329 10.1164/rccm.200209-1112OC

[b8] Hogan B. L. Morphogenesis. Cell. 96, 225–233 (1999). 9988217 10.1016/s0092-8674(00)80562-0

[b9] Shannon J. M. Induction of alveolar type II cell differentiation in fetal tracheal epithelium by grafted distal lung mesenchyme. Dev Biol. 166, 600–614 (1994). 7813779 10.1006/dbio.1994.1340

[b10] Shannon J. M., Nielsen L. D., Gebb S. A. & Randell S. H. Mesenchyme specifies epithelial differentiation in reciprocal recombinants of embryonic lung and trachea. Dev Dyn. 212, 482–494 (1998). 9707322 10.1002/(SICI)1097-0177(199808)212:4<482::AID-AJA2>3.0.CO;2-D

[b11] Hogan B. L. *et al.* Repair and regeneration of the respiratory system: complexity, plasticity, and mechanisms of lung stem cell function. Cell Stem Cell. 15, 123–138 (2014). 25105578 10.1016/j.stem.2014.07.012 PMC4212493

[b12] Bellusci S., Grindley J., Emoto H., Itoh N. & Hogan B. L. Fibroblast growth factor 10 (FGF10) and branching morphogenesis in the embryonic mouse lung. Development. 124, 4867–4878 (1997). 9428423 10.1242/dev.124.23.4867

[b13] Hogan B. L. *et al.* Branching morphogenesis of the lung: new models for a classical problem. Cold Spring Harb Symp Quant Biol. 62, 249–256 (1997). 9598358

[b14] Warburton D. *et al.* The molecular basis of lung morphogenesis. Mech Dev. 92, 55–81 (2000). 10704888 10.1016/s0925-4773(99)00325-1

[b15] Cardoso W. V. Transcription factors and pattern formation in the developing lung. Am J Physiol. 269, L429–L442 (1995). 7485515 10.1152/ajplung.1995.269.4.L429

[b16] Hrycaj S. M. *et al.* Hox5 Genes Regulate the Wnt2/2b-Bmp4-Signaling Axis during Lung Development. Cell Rep. 12, 903–912 (2015). 26235626 10.1016/j.celrep.2015.07.020 PMC4536095

[b17] Goss A. M. *et al.* Wnt2 signaling is necessary and sufficient to activate the airway smooth muscle program in the lung by regulating myocardin/Mrtf-B and Fgf10 expression. Dev Biol. 356, 541–552 (2011). 21704027 10.1016/j.ydbio.2011.06.011 PMC3319016

[b18] Valcourt U., Thuault S., Pardali K., Heldin C. H. & Moustakas A. Functional role of Meox2 during the epithelial cytostatic response to TGF-beta. Mol Oncol. 1, 55–71 (2007). 19383287 10.1016/j.molonc.2007.02.002 PMC5543849

[b19] Chen Y., Rabson A. B. & Gorski D. H. MEOX2 regulates nuclear factor-kappaB activity in vascular endothelial cells through interactions with p65 and IkappaBbeta. Cardiovasc Res. 87, 723–731 (2010). 20421348 10.1093/cvr/cvq117 PMC2920806

[b20] Chen Y. *et al.* Regulation of the expression and activity of the antiangiogenic homeobox gene GAX/MEOX2 by ZEB2 and microRNA-221. Mol Cell Biol. 30, 3902–3913 (2010). 20516212 10.1128/MCB.01237-09 PMC2916411

[b21] Cortese R., Hartmann O., Berlin K. & Eckhardt F. Correlative gene expression and DNA methylation profiling in lung development nominate new biomarkers in lung cancer. Int J Biochem Cell Biol. 40, 1494–1508 (2008). 18203646 10.1016/j.biocel.2007.11.018

[b22] Warburton D. Developmental responses to lung injury: repair or fibrosis. Fibrogenesis Tissue Repair. 5, S2 (2012). 23259863 10.1186/1755-1536-5-S1-S2 PMC3368777

[b23] Kugler M. C., Joyner A. L., Loomis C. A. & Munger J. S. Sonic hedgehog signaling in the lung. From development to disease. Am J Respir Cell Mol Biol. 52, 1–13 (2015). 25068457 10.1165/rcmb.2014-0132TR PMC4370254

[b24] Gupte V. V. *et al.* Overexpression of fibroblast growth factor-10 during both inflammatory and fibrotic phases attenuates bleomycin-induced pulmonary fibrosis in mice. Am J Respir Crit Care Med. 180, 424–436 (2009). 19498056 10.1164/rccm.200811-1794OC PMC2742760

[b25] McQualter J. L. *et al.* TGF-beta signaling in stromal cells acts upstream of FGF-10 to regulate epithelial stem cell growth in the adult lung. Stem Cell Res. 11, 1222–1233 (2013). 24029687 10.1016/j.scr.2013.08.007

[b26] Upadhyay D., Bundesmann M., Panduri V., Correa-Meyer E. & Kamp D. W. Fibroblast growth factor-10 attenuates H2O2-induced alveolar epithelial cell DNA damage: role of MAPK activation and DNA repair. Am J Respir Cell Mol Biol. 31, 107–113 (2004). 14975937 10.1165/rcmb.2003-0064OC

[b27] Cunnington R. H. *et al.* The Ski-Zeb2-Meox2 pathway provides a novel mechanism for regulation of the cardiac myofibroblast phenotype. J Cell Sci. 127, 40–49 (2014). 24155330 10.1242/jcs.126722

[b28] Stappenbeck T. S. & Miyoshi H. The role of stromal stem cells in tissue regeneration and wound repair. Science. 324, 1666–1669 (2009). 19556498 10.1126/science.1172687

[b29] Lama V. N. *et al.* Evidence for tissue-resident mesenchymal stem cells in human adult lung from studies of transplanted allografts. J Clin Invest. 117, 989–996 (2007). 17347686 10.1172/JCI29713 PMC1810571

[b30] Irizarry R. A. *et al.* Exploration, normalization, and summaries of high density oligonucleotide array probe level data. Biostatistics. 4, 249–264 (2003). 12925520 10.1093/biostatistics/4.2.249

[b31] Smyth G. K. Linear models and empirical bayes methods for assessing differential expression in microarray experiments. Stat Appl Genet Mol Biol. 3, Article3 (2004). 16646809 10.2202/1544-6115.1027

[b32] Thomas S. & Bonchev D. A survey of current software for network analysis in molecular biology. Hum Genomics. 4, 353–360 (2010). 20650822 10.1186/1479-7364-4-5-353 PMC3500165

[b33] Fernandez I. E. & Eickelberg O. The impact of TGF-beta on lung fibrosis: from targeting to biomarkers. Proc Am Thorac Soc. 9, 111–116 (2012). 22802283 10.1513/pats.201203-023AW

[b34] Bolanos A. L. *et al.* Role of Sonic Hedgehog in idiopathic pulmonary fibrosis. Am J Physiol Lung Cell Mol Physiol. 303, L978–L990 (2012). 23023967 10.1152/ajplung.00184.2012

[b35] Herriges J. C. *et al.* FGF-Regulated ETV Transcription Factors Control FGF-SHH Feedback Loop in Lung Branching. Dev Cell. 35, 322–332 (2015). 26555052 10.1016/j.devcel.2015.10.006 PMC4763945

[b36] Hardie W. D. *et al.* Signaling pathways in the epithelial origins of pulmonary fibrosis. Cell Cycle. 9, 2769–2776 (2010). 20676040 10.4161/cc.9.14.12268 PMC3040960

[b37] Kumar M. E. *et al.* Mesenchymal cells. Defining a mesenchymal progenitor niche at single-cell resolution. Science. 346, 1258810 (2014). 25395543 10.1126/science.1258810 PMC4269943

[b38] Sanders Y. Y. *et al.* Altered DNA methylation profile in idiopathic pulmonary fibrosis. Am J Respir Crit Care Med. 186, 525–535 (2012). 22700861 10.1164/rccm.201201-0077OC PMC3480526

[b39] Sanders Y. Y., Liu H., Liu G. & Thannickal V. J. Epigenetic mechanisms regulate NADPH oxidase-4 expression in cellular senescence. Free Radic Biol Med. 79, 197–205 (2015). 25526894 10.1016/j.freeradbiomed.2014.12.008

[b40] Volckaert T. & De Langhe S. Lung epithelial stem cells and their niches: Fgf10 takes center stage. Fibrogenesis Tissue Repair. 7, 8 (2014). 24891877 10.1186/1755-1536-7-8 PMC4041638

[b41] Min H. *et al.* Fgf-10 is required for both limb and lung development and exhibits striking functional similarity to Drosophila branchless. Genes Dev. 12, 3156–3161 (1998). 9784490 10.1101/gad.12.20.3156 PMC317210

[b42] Volckaert T. *et al.* Parabronchial smooth muscle constitutes an airway epithelial stem cell niche in the mouse lung after injury. J Clin Invest. 121, 4409–4419 (2011). 21985786 10.1172/JCI58097 PMC3204843

[b43] Tong L. *et al.* Fibroblast Growth Factor-10 (FGF-10) Mobilizes Lung-resident Mesenchymal Stem Cells and Protects Against Acute Lung Injury. Sci Rep. 6, 21642 (2016). 26869337 10.1038/srep21642 PMC4751498

[b44] Li C. *et al.* Wnt5a regulates Shh and Fgf10 signaling during lung development. Dev Biol. 287, 86–97 (2005). 16169547 10.1016/j.ydbio.2005.08.035

[b45] Lebeche D., Malpel S. & Cardoso W. V. Fibroblast growth factor interactions in the developing lung. Mech Dev. 86, 125–136 (1999). 10446271 10.1016/s0925-4773(99)00124-0

[b46] Hu B. *et al.* Reemergence of hedgehog mediates epithelial-mesenchymal crosstalk in pulmonary fibrosis. Am J Respir Cell Mol Biol. 52, 418–428 (2015). 25140582 10.1165/rcmb.2014-0108OC PMC4491120

[b47] Fitch P. M., Howie S. E. & Wallace W. A. Oxidative damage and TGF-beta differentially induce lung epithelial cell sonic hedgehog and tenascin-C expression: implications for the regulation of lung remodelling in idiopathic interstitial lung disease. Int J Exp Pathol. 92, 8–17 (2011). 21039988 10.1111/j.1365-2613.2010.00743.x PMC3052752

[b48] Pepicelli C. V., Lewis P. M. & McMahon A. P. Sonic hedgehog regulates branching morphogenesis in the mammalian lung. Curr Biol. 8, 1083–1086 (1998). 9768363 10.1016/s0960-9822(98)70446-4

[b49] Petrova R. & Joyner A. L. Roles for Hedgehog signaling in adult organ homeostasis and repair. Development. 141, 3445–3457 (2014). 25183867 10.1242/dev.083691 PMC4197719

[b50] Peng T. *et al.* Hedgehog actively maintains adult lung quiescence and regulates repair and regeneration. Nature. (2015). 10.1038/nature14984 PMC4713039 26436454

[b51] van Tuyl M. & Post M. From fruitflies to mammals: mechanisms of signalling via the Sonic hedgehog pathway in lung development. Respir Res. 1, 30–35 (2000). 11667962 10.1186/rr9 PMC59539

[b52] Warburton D. *et al.* Molecular mechanisms of early lung specification and branching morphogenesis. Pediatr Res. 57, 26R–37R (2005). 10.1203/01.PDR.0000159570.01327.ED 15817505

[b53] Weaver M., Dunn N. R. & Hogan B. L. Bmp4 and Fgf10 play opposing roles during lung bud morphogenesis. Development. 127, 2695–2704 (2000). 10821767 10.1242/dev.127.12.2695

[b54] Upton P. D., Davies R. J., Tajsic T. & Morrell N. W. Transforming growth factor-beta(1) represses bone morphogenetic protein-mediated Smad signaling in pulmonary artery smooth muscle cells via Smad3. Am J Respir Cell Mol Biol. 49, 1135–1145 (2013). 23937428 10.1165/rcmb.2012-0470OC PMC3931109

[b55] Leask A. & Abraham D. J. TGF-beta signaling and the fibrotic response. FASEB J. 18, 816–827 (2004). 15117886 10.1096/fj.03-1273rev

[b56] Khalil N., O’Connor R. N., Flanders K. C. & Unruh H. TGF-beta 1, but not TGF-beta 2 or TGF-beta 3, is differentially present in epithelial cells of advanced pulmonary fibrosis: an immunohistochemical study. Am J Respir Cell Mol Biol. 14, 131–138 (1996). 8630262 10.1165/ajrcmb.14.2.8630262

[b57] Broekelmann T. J., Limper A. H., Colby T. V. & McDonald J. A. Transforming growth factor beta 1 is present at sites of extracellular matrix gene expression in human pulmonary fibrosis. Proc Natl Acad Sci USA 88, 6642–6646 (1991). 1862087 10.1073/pnas.88.15.6642 PMC52144

[b58] Wahl S. M. Transforming growth factor beta: the good, the bad, and the ugly. J Exp Med. 180, 1587–1590 (1994). 7964446 10.1084/jem.180.5.1587 PMC2191721

[b59] Volckaert T. & De Langhe S. P. Wnt and FGF mediated epithelial-mesenchymal crosstalk during lung development. Dev Dyn. 244, 342–366 (2015). 25470458 10.1002/dvdy.24234 PMC4344844

[b60] Hecker L. *et al.* NADPH oxidase-4 mediates myofibroblast activation and fibrogenic responses to lung injury. Nat Med. 15, 1077–1081 (2009). 19701206 10.1038/nm.2005 PMC2743335

