# Supplementary material for: Developmental Reprogramming in Mesenchymal Stromal Cells of Human Subjects with Idiopathic Pulmonary Fibrosis
Source: Sci Rep. 2016 Nov 21;6:37445. doi: 10.1038/srep37445 (PMC5116673; doi:10.1038/srep37445)
Supplement: Supplementary Information [file srep37445-s1.pdf]

Developmental Reprogramming in Mesenchymal Stromal Cells of Human Subjects with Idiopathic Pulmonary Fibrosis

Diptiman Chanda[1*], Ashish Kurundkar[1], Sunad Rangarajan[1], Morgan Locy[1], Karen Bernard[1], Nirmal S. Sharma[1], Naomi J. Logsdon[1], Hui Liu[1], David K. Crossman[2], Jeffrey C. Horowitz[3], Stijn De Langhe[4], and Victor J. Thannickal[1*]

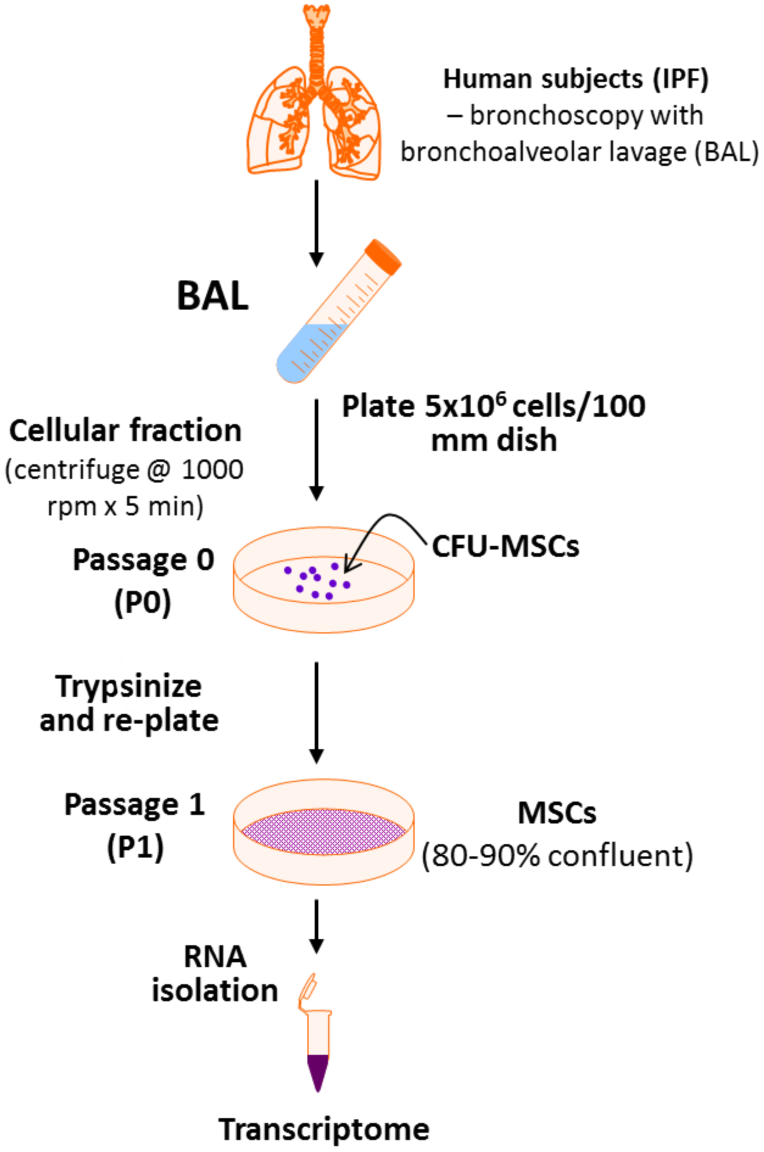

Supplementary Figure S1: Protocol for isolation of mesenchymal stromal cells (MSCs) from human subjects with IPF. IPF subjects underwent bronchoscopy with bronchoalveolar lavage (BAL) and recovered fluid subjected to centrifugation and re-suspension of the cell pellet in media prior to plating and *ex vivo* culture as described in Methods; RNA for Affymetrix analysis was isolated from MSCs at passage 2.

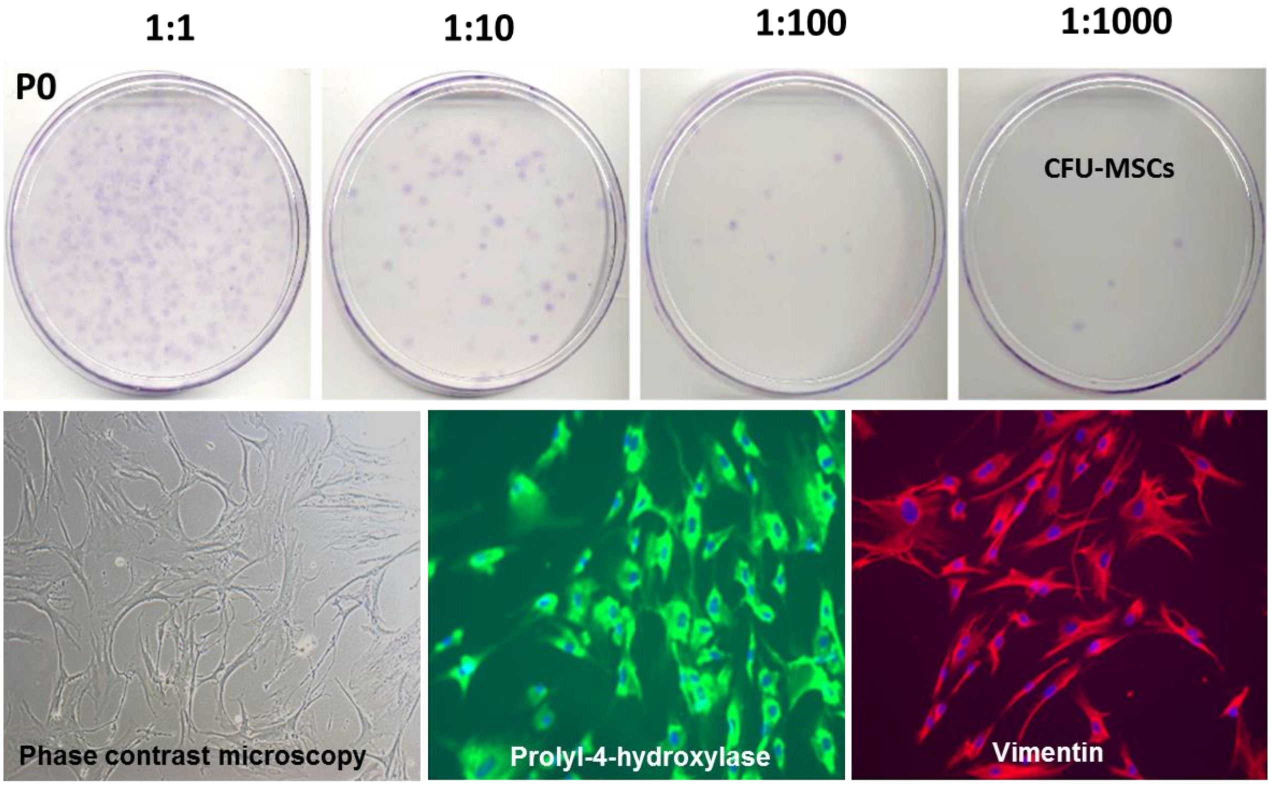

Supplementary Figure S2: **Morphology and mesenchymal phenotype of MSCs**. Top panels, MSCs plated from re-suspended cell pellet following BAL fluid centrifugation; colony-forming units (CFU-MSCs; Giemsa stain) are shown at serial dilutions. Lower panels, MSCs display fibroblastoid morphology and uniformly stain for the mesenchymal markers, prolyl-4-hydroxylase and vimentin.

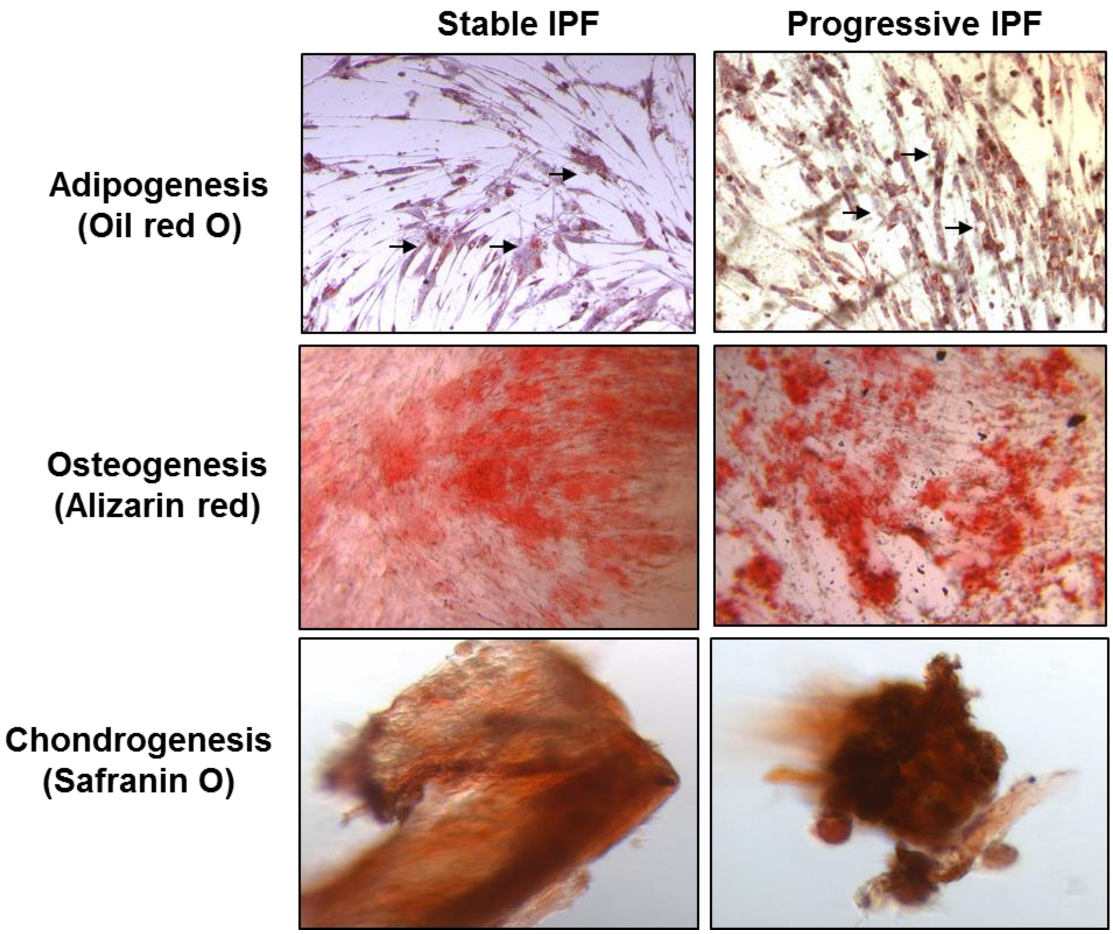

Supplementary Figure S3: **Mesodermal lineage differentiation of BAL-MSCs obtained from stable and progressive IPF patients**. Early passage MSCs were grown in 24-well dishes and cultured in either adipocyte or osteogenic differentiation medium for 10-14 days. Lipid granules in the adipocytes were visualized with Oil Red O stain (arrows) whereas calcium depositions in the newly formed osteoblasts were visualized with Alizarin Red stain. For chondrogenesis early passage MSCs were pelleted in conical bottom tubes and maintained in chondrogenic differentiation medium for 14 days. The cell pellet was fixed in neutral buffered formalin and stained with Safranin-O to visualize proteoglycans within the newly formed chondrocytes (original magnification 200x).

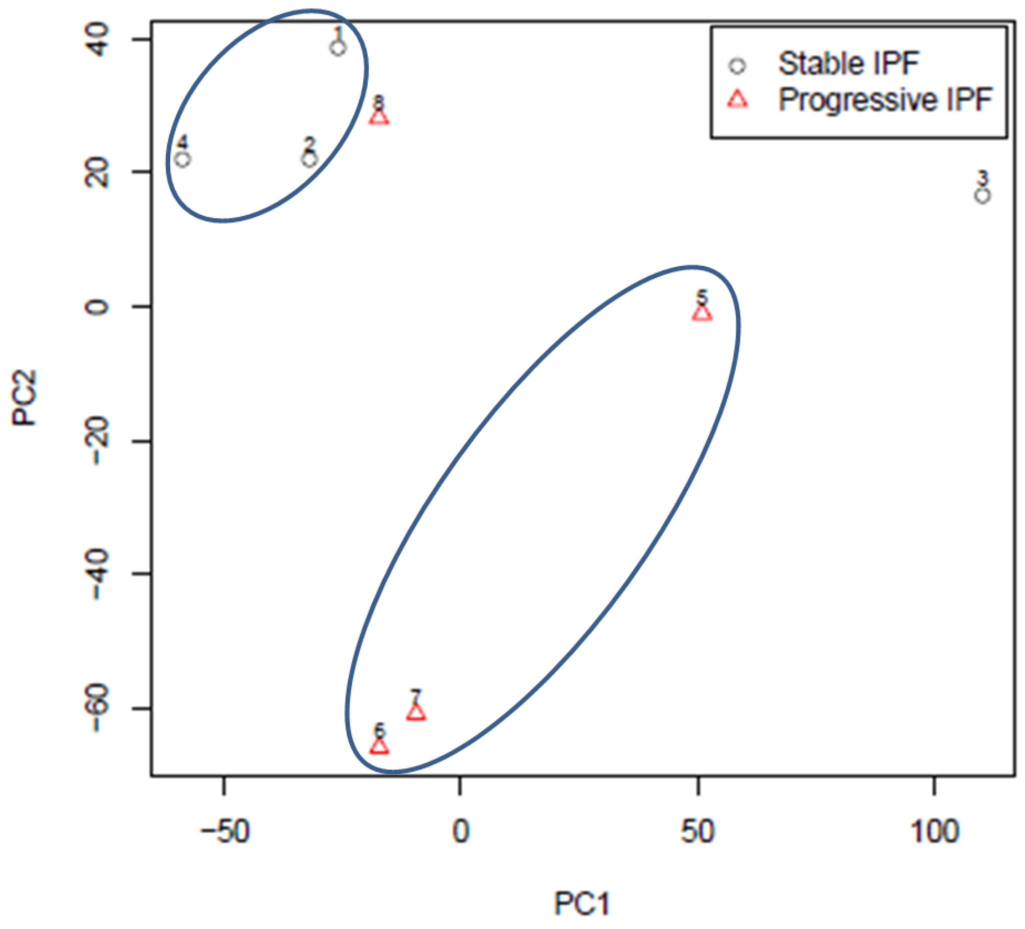

Supplementary Figure S4: **Principal Components Analysis** (**PCA**) **of gene expression values**. PCA was used to visualize the overall structure of the high dimensional data which determines if the replicated samples (stable IPF and progressive IPF) are relatively similar in their gene expression profiles. Based on PCA, sample # 3 and sample # 8 were excluded from gene network analysis.

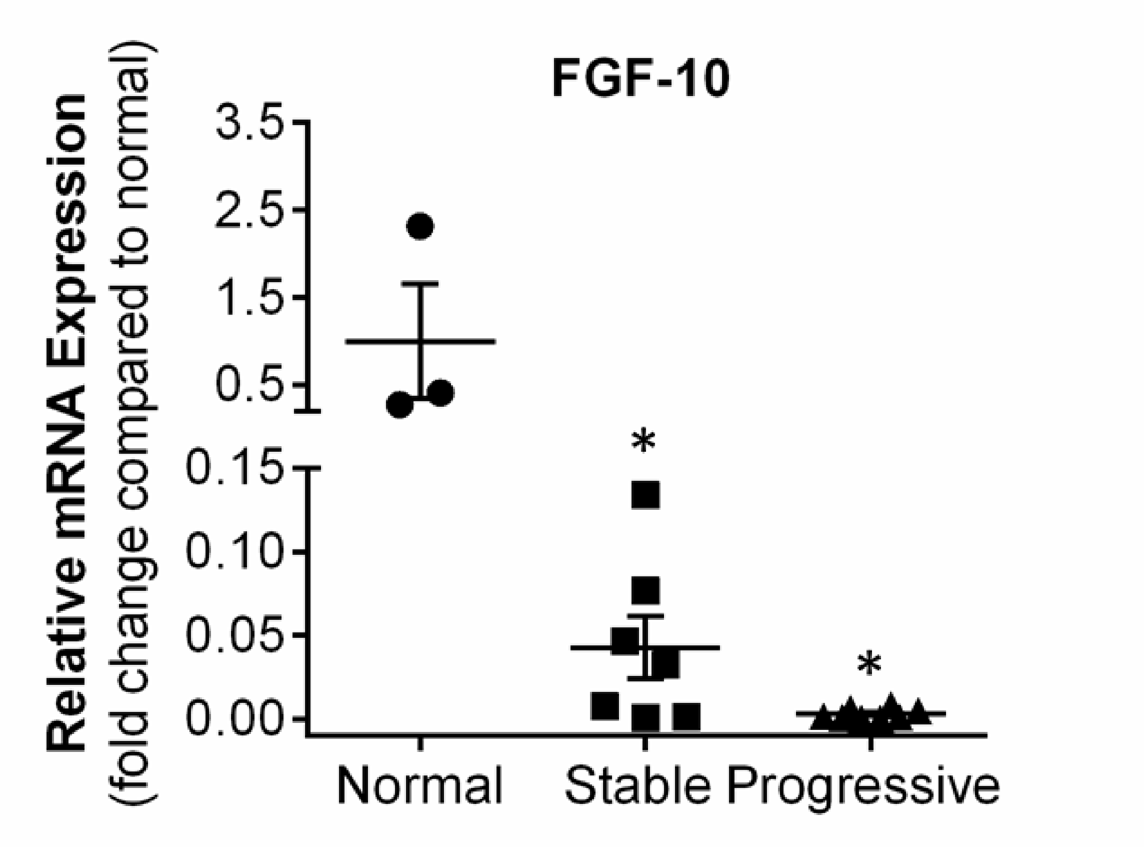

Supplementary Figure S5: Total RNA was isolated from MSCs, isolated from BAL of lung transplant recipients (n=3), stable IPF (n=7) and progressive IPF (n=8), and subjected to real-time PCR analysis for FGF-10. Data were normalized to 18S rRNA and represented graphically as fold change compared to stable IPF (s-IPF). FGF-10 gene expression in normal MSCs isolated from lung transplant recipients were significant higher (*$p < 0.03$) compared to MSCs isolated from BAL of IPF subjects.

A

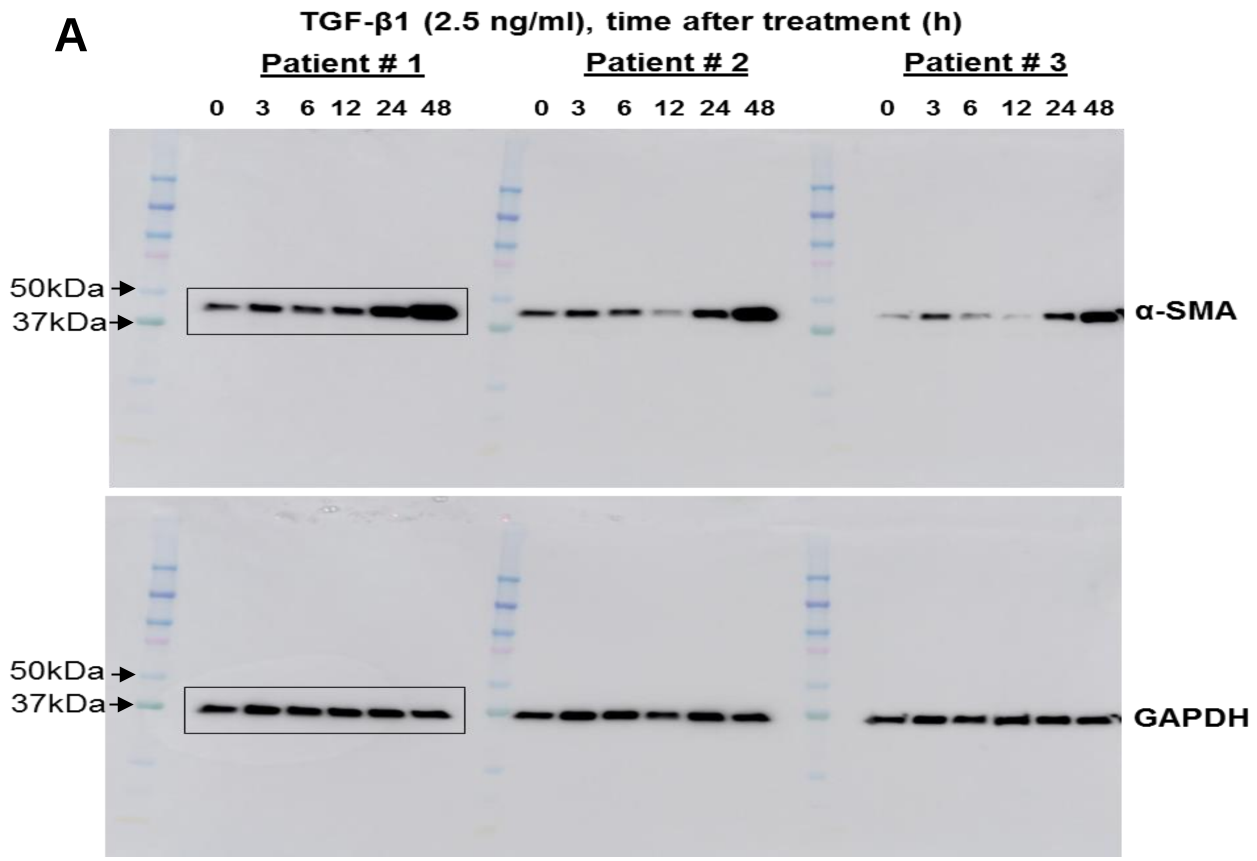

TGF-β1 (2.5 ng/ml), time after treatment (h)
Patient # 1
Patient # 2
Patient # 3
0 3 6 12 24 48
0 3 6 12 24 48
0 3 6 12 24 48
50kDa
37kDa
α-SMA
50kDa
37kDa
GAPDH

B

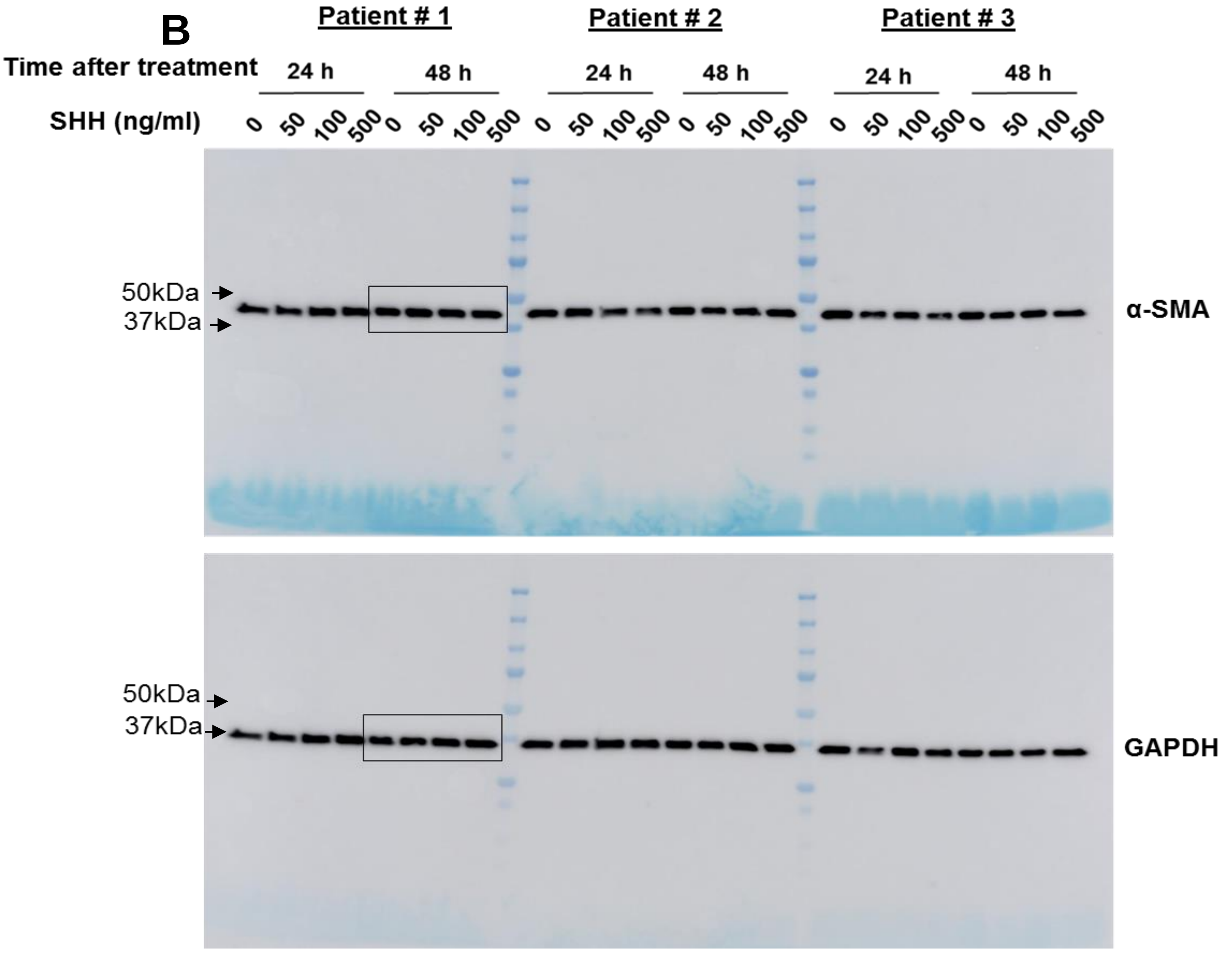

Patient # 1
Patient # 2
Patient # 3
Time after treatment
24 h
48 h
24 h
48 h
24 h
48 h
SHH (ng/ml)
0 50 100 500 0 50 100 500
0 50 100 500 0 50 100 500
0 50 100 500 0 50 100 500
50kDa
37kDa
α-SMA
50kDa
37kDa
GAPDH

Supplementary Figure S6: Effects of TGF-β1 (A) and SHH (B) on myofibroblast differentiation of BAL-derived MSCs. MSCs were isolated from surveillance bronchoscopies and BAL from lung transplant recipients without bronchiolitis obliterans or infection. MSCs were seeded in 6-well tissue culture plates and serum deprived for 24 h followed by either TGF-β1 treatment (2.5 ng/ml) or SHH (0, 50, 100, 500 ng/ml) for 48 h. Cell lysates were prepared in RIPA buffer and subjected to SDS-PAGE and western blot analysis for α-SMA; GAPDH antibody was used as loading control. The protein bands outlined here are presented in **Figure 4 A&B**. Densitometry was performed to quantitate the ratio of α-SMA and GAPDH and plotted graphically (n = 3).

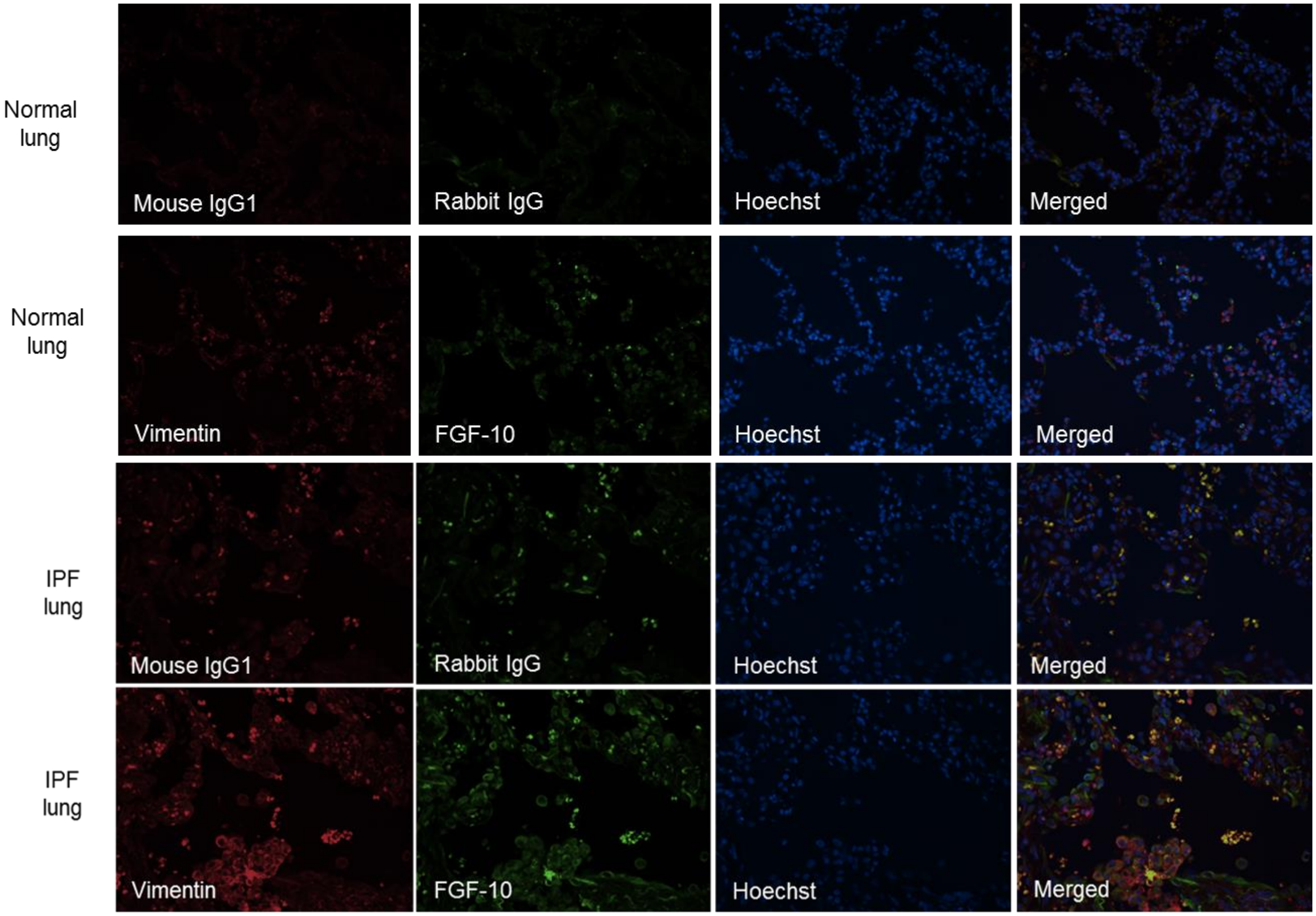

Supplementary Figure S7: **Immunofluorescence staining for vimentin and FGF-10 in normal and IPF lungs**. Six micron (6µ) sections were obtained from normal (n=4; failed donor lungs) and IPF (n=4; explants during lung transplantation) lungs. In IPF lung tissue, FGF-10 expression was observed in the interstitial mesenchymal cells in regions with less fibrotic remodeling. FGF-10 expression in these cells also co-localized with MSC marker, vimentin. Although vimentin expression was detected in the interstitial mesenchymal cells in normal lungs, FGF-10 expression was largely undetected in normal lungs. IgG isotype controls were used to determine specificity of the FGF-10 and vimentin antibodies (Original magnification X400).

Rank	Gene Function	Score	Focus Molecules	Molecules in Network	
				Up-regulated	Down-regulated
1	Cellular Assembly and Organization, Cell Cycle, DNA Replication, Recombination, and Repair	41	24	ADCY7, BUB1, BUB1B, CASC5, CDC6, CDC20, CDKN3, CENPE, LOX, NDC80, NFIB, NUF2, NUSAP1, OIP5, PVR, RRAS2	C1R, CDKN1C, DLEU2, GUC1A3, GUCY1B3, RARRES3, ST8SIA1, VSNL1
2	Organismal Development, Cellular Growth and Proliferation, Cardiovascular System Development and Function	37	23	ANXA3, CNN1, COL4A1, CYR61, F3, F11R, GATA2, HMMR, ID4, ITGA6, **MEOX2**, RCAN1, SEMA3F, SERPINE1	**BMP4**, COL18A1, **FGF10**, GREM2, IGFBP2, MMP11, MTUS1, PERP, SHC3
3	Cell Cycle, Cellular Assembly and Organization, DNA Replication, Recombination, and Repair	34	23	CCNA2, CCNB1, CCNB2, CDC25C, CDK1, CDK18, DLGAP5, EBP, KIF14, KIF23, KIF2C, MAD2L1, NEK2, PBK, PNMA2, PRC1, PTTG1, RAD51AP1, SHCBP1, TTK, UBE2C, UHRF1, VAMP8	
4	Carbohydrate Metabolism, Small Molecule Biochemistry, Cancer	34	21	BIRC5, CD36, LIPG, PDCD6, PDE1C, PDK1, PRKAG2, UCP2	CLU, CPE, DUSP4, FBXO32, GAS1, IL1R1, PDE5A, PDE7B, PDK4, PPARGC1A, PTN, SNCAIP, TNFSF10
5	Cell Cycle, Cellular Assembly and Organization, DNA Replication, Recombination, and Repair	32	20	ASPM, CDCA3, CDKN3, CENPE, **HOXA2**, KCNK1, MATN3, NCAPG (includes EG:64151), NDC80, NFIB, PRPS1, PSG1, SH2D5, SPINT2	COL14A1, GAS1, MYO10, NOVA1, PLXNC1, TOX

Supplementary Table S1: **Gene ontology networks identified by ingenuity pathways analysis**. Total RNA was isolated from MSCs from s-IPF and p-IPF and transcriptomic analysis performed by Affymetrix arrays. Genes that were significantly up-regulated or down-regulated (n = 3 in each group; 2-fold change, $p < 0.005$) were subjected to ingenuity pathway analysis (IPA). The top functioning networks, related IPA score for each network and the specific network genes that were either up- or down-regulated are shown. The score is based on a *p*-value calculation that defines the likelihood that "network eligible" molecules that are part of the network are found therein by random chance alone. Depicted in bold are developmental genes (FGF-10, BMP-4, Meox2 and HoxA2) that were selected for further analysis.

Gene Name and Accession Number		Primer Sequence (5’-3’)	Product Size (bp)	Annealing/ Extension Temperature (ºC)
**FGF-10 NM_004465.1**	Forward	ATGTCCGCTGGAGAAAGCTA	188	60
	Reverse	CCCCTTCTTGTTCATGGCTA		
**BMP-4 NM_001202.3**	Forward	TGATACCTGAGACGGGGAAG	199	60
	Reverse	CCAGACTGAAGCCGGTAAAG		
**Meox2 NM_005924.4**	Forward	TCTCACCAGACTGAGGCGAT	118	66
	Reverse	TTGCTGTCCACCCTTTACCC		
**HoxA2 NM_006735.3**	Forward	TTCAGCAAAATGCCCTCTCT	176	60
	Reverse	TAGGCCAGCTCCACAGTTCT		
**18S rRNA X03205.1**	Forward	GTCTGCCCTATCAACTTTCG	111	60
	Reverse	ATGTGGTAGCCGTTTCTCA		

Supplementary Table S2: List of PCR primers used in this study and conditions
